# Barriers and Facilitators to the Implementation of a Community Doula Program for Black and Pacific Islander Pregnant People in San Francisco: Findings from a Partnered Process Evaluation

**DOI:** 10.1007/s10995-022-03373-x

**Published:** 2022-01-24

**Authors:** Cassondra Marshall, Stephanie Arteaga, Jennet Arcara, Alli Cuentos, Marna Armstead, Andrea Jackson, Anu Manchikanti Gómez

**Affiliations:** 1 School of Public Health, University of California, Berkeley, 2121 Berkeley Way #5302, Berkeley, CA 94720-7360 USA; 2 Sexual Health and Reproductive Equity Program, School of Social Welfare, University of California, Berkeley, 110 Haviland Hall, MC 7400, Berkeley, CA 94720-7400 USA; 3 SisterWeb San Francisco Community Doula Network, 1912 Keith St., San Francisco, CA 94124 USA; 4 Department of Obstetrics, Gynecology, and Reproductive Sciences, University of California, San Francisco, 2356 Sutter St. J-140, San Francisco, CA 94115 USA

**Keywords:** Process evaluation, Doulas, Black birthing people, Pacific Islander birthing people, Community health worker

## Abstract

**Introduction:**

Increasingly, community-based models of doula care are receiving attention as possible interventions to address racial inequities in maternal health care experiences and outcomes. In 2018, community-based organization SisterWeb launched to provide free culturally congruent community doula care to advance birth equity for Black and Pacific Islander pregnant people, with funding from the San Francisco Department of Public Health. We conducted a process evaluation of SisterWeb’s first 1.5 years of existence to identify multilevel barriers and facilitators to implementation of their programs.

**Methods:**

Guided by the Equitable Evaluation Framework™, we conducted 46 in-depth interviews with individuals from 5 groups: SisterWeb leadership, doulas, doula mentors, and clients, and external stakeholders.

**Results:**

Barriers included having diverse clientele groups with unique needs, an ineffective payment model, and simultaneously building an organization and developing and implementing programs. Facilitators included the presence of established strategic partnerships, positive reception of services due to an unmet need for culturally and linguistically congruent pregnancy and birth support among SisterWeb’s clients, a clear organizational vision and mission, and a unique doula cohort model.

**Discussion:**

Our findings suggest developing community doula programs pay close attention to the difference between launching a *program* versus an *organization*, including the required resources of each, the sustainability of payment models for community doulas, and the provision of culturally relevant, needed services within priority communities. Furthermore, strategic partnerships with maternal health stakeholders in birthing sites, particularly hospitals, are vital to the success of a community doula program.

## Significance

“What is already known on this subject?”

Doula care is an evidence-based intervention to improve maternal health, patient satisfaction, and health care experiences. *Community* doula care is increasingly recognized as an intervention to address racial inequities in maternal and infant health and health care, but research on community doula care is scant.

“What this study adds?”

Using findings from a robust process evaluation, this study identifies multilevel barriers and facilitators to the implementation of two community-based, culturally concordant doula programs serving Black and Pacific Islander pregnant people.

## Introduction

Despite its developed economy and advanced infrastructure, the United States (US) continues to face a persistent maternal and infant health crisis disproportionately affecting Black, Indigenous, and people of color and people with low incomes (Tikkanen et al., 2020). The US has the highest rate of maternal and infant mortality compared to other developed nations; in 2018, there were 17.4 maternal deaths in the United States per 100,000 live births, a rate two times or more higher than other developed nations (Tikkanen et al., 2020). Black women experience the worst maternal health outcomes compared to any other racial or ethnic group in the country, including the highest rates of preterm birth (Ely et al., 2018; Martin et al., 2018a, 2018b; Singh & Yu, 2019). In California, evidence suggests that Pacific Islander mothers and their infants also experience poor maternal and infant health outcomes, similar to those of Black women and infants (California Department of Public Health, 2020; Jelliffe-Pawlowski et al., 2015).

These long standing maternal and infant health disparities stem, in part, from poor quality of maternity care. Women of color are more likely to report negative interactions with practitioners and mistreatment during maternity care and, subsequently, dissatisfaction with birth experiences compared to white women (Sakala et al., 2018; Vedam et al., 2019). Specifically, women have reported being treated unfairly because of their race, ethnicity, or language, being spoken to with harsh or rude language, or being handled roughly by maternity care providers (Sakala et al., 2018; Vedam et al., 2019). These experiences do not align with quality maternal care, which is defined by the World Health Organization as care that is “safe, effective, timely, efficient, equitable, and people-centered” (World Health Organization, 2016). Importantly, these negative interactions with healthcare providers are influenced by historical experiences of racism in healthcare, leading to a lack of trust in healthcare providers among people of color, and racial biases among healthcare providers (Gamble, 1997; Gómez & Wapman, 2017; Haider et al., 2013; Roberts, 1999; Sacks, 2019).

There has been increasing recognition of doula care as an evidence-based intervention to improve maternal health, patient satisfaction, and health care experiences (Bohren et al., 2017; Kozhimannil et al., Kozhimannil, Attanasio, et al., 2013; Kozhimannil et al., 2014). A doula is a trained professional who provides nonclinical support to pregnant people before, during, and after birth; some doulas also provide support for abortions, miscarriage, and infant death (Bey et al., 2019). A 2017 Cochrane review reported that women receiving continuous labor support, such as that provided by doulas, were more likely to have spontaneous vaginal births and less likely to report dissatisfaction with their birth experiences, to have caesarean sections, and to have instrumental vaginal births (Bohren et al., 2017). Furthermore, the existing literature on the benefits of doula care suggest that doula support can also lead to lower rates of preterm birth (Kozhimannil et al., Kozhimannil et al., 2016; Kozhimannil, Attanasio, et al., 2013; Thomas et al., 2017), low birth weight (Gruber et al., 2013), epidural use (Paterno et al., 2012), and birth complications (Gruber et al., 2013). Research also suggests that doula care can lead to higher rates of breastfeeding initiation (Gruber et al., 2013; Hans et al., 2018; Kozhimannil, Attanasio, et al., 2013; Kozhimannil, Johnson, et al., 2013; Mottl-Santiago et al., 2008), safe sleep and car seat safety practices (Hans et al., 2018), attending childbirth classes (Hans et al., 2018), and utilization of non-medical pain management during childbirth (Kozhimannil et al., Kozhimannil, Attanasio, et al., 2013). Potential cost-savings are associated with doula-assisted births as a result of the lower rates of costly outcomes (Chapple et al., 2013; Kozhimannil et al., 2016), prompting a commentary advocating for health insurance coverage of doula services, particularly for low-income women (Strauss et al., 2016). In light of the ongoing maternal health crisis, policymakers across the country are considering expanding access to doula services (Patel & Chen, 2020).

More recently, there has been a focus on *community* doula care as an intervention to address inequitable maternal and infant health outcomes among women of color. Community doulas are typically members of the community they serve, and therefore share experiences with their clients that allow them to better address the unique issues they face, including racial discrimination and language barriers (Bey et al., 2019). Usually, this care is provided at low or no cost to enable access to the service for people who need them most (Bey et al., 2019). Community-based models of doula care have been recognized as a key strategy for ending racial disparities in maternal health experiences and outcomes (Bey et al., 2019; New York State Taskforce on Maternal Mortality and Disparate Racial Outcomes, 2019). Notably, much existing research on doula care focuses on private doula care models (in which clients pay out-of-pocket) and the clients who can afford such care; research on community doula care, specifically, and its impact on the populations served, is scant.

In 2018, Expecting Justice, a cross-sector initiative of the San Francisco Department of Public Health, other city agencies, community-based organizations, health providers, and community members, identified doula care for Black and Pacific Islander pregnant people in San Francisco as a strategy for advancing birth equity and partnered with a community-based organization, the SisterWeb San Francisco Community Doula Network, to launch an initiative providing these services at no-cost. Expecting Justice focuses on Black and Pacific Islander communities because they experience the highest rates of preterm birth in San Francisco (Expecting Justice, n.d.; San Francisco Department of Public Health, 2018). The purpose of this paper is to describe the key barriers and facilitators to the implementation of SisterWeb’s programs. These findings can inform the development, implementation, and evaluation of other community doula interventions to advance maternal and infant health equity.

### Organization Description

In their own words, SisterWeb works to build a San Francisco where “Black, Pacific Islander, and Latina/o/x families…are centered and uplifted in their reproductive journey to welcome their children with respect, dignity, joy and pride, leading to thriving families and communities and increased birth equity and justice” (*Our Story | SisterWeb*, 2021). Founded in 2018, SisterWeb works towards its mission in two ways: by providing no-cost, high quality doula care to birthing people of color most at risk for adverse birth outcomes, and by providing their community doulas—who are from the communities they serve—with stable employment and the tools, job skills, and mentorship needed to succeed as professional birth workers.

### Doula Programs

SisterWeb has three programmatic arms: Kindred Birth Companions, serving Black clients; M.A.N.A. Pasefika, serving Pacific Islander clients; and Semilla Sagrada, serving Latinx clients. To aid in providing community doula services, SisterWeb has developed partnerships with four of the five hospitals with labor and delivery units in San Francisco, and the only free-standing birth center. Client referrals to SisterWeb come from medical providers, social workers or others at community-based services, and directly from prospective clients themselves via SisterWeb’s website.

SisterWeb utilizes a cohort model for their doulas, consisting of two to three doulas and two doula mentors per cohort to avoid doula burnout and ensure their clients always have access to a doula. Due to the low number of Pacific Islander births in San Francisco and reduced funding, the M.A.N.A Pasefika program currently only has one doula. All three programs follow the same model of care. Typically, SisterWeb doulas begin their work with mothers between 10 and 27 weeks of pregnancy (although clients later in pregnancy are accepted when there is space), and their model of care includes a total of 8 visits that include an initial intake visit and three prenatal visits, in-person labor and birth support, and four postpartum visits. Clients needing extra support are internally designated “Code Purple”; these clients have more interaction with doulas and are connected to additional community resources. Table 1 describes key elements of SisterWeb’s programs. Since its launch, the M.A.N.A. Pasefika program has shifted to a model including community engagement activities with San Francisco’s Pacific Islander community to increase participation in the program. In March 2020, doulas were temporarily unable to provide in-person services due to the onset of the COVID-19 pandemic, but since July 2020, have largely returned to providing in-person birth and labor support. Currently, prenatal and postpartum visits are held over phone or videoconferencing.

**Table 1:** Key components of SisterWeb’s program

Component	Description
Cohort Model	SisterWeb doulas and mentors work in cohorts, comprised of two or three doulas. Each client is assigned a doula cohort. Clients develop relationships with all doulas in their assigned cohort, so that depending on when support is needed, a trusted and known person is there.
Mentorship Model	Cohorts also include 1-2 mentors, who provide support and guidance for doulas in their cohort. Mentors are focused on supporting the doulas' birth knowledge goals/proficiency as well as supporting clients with complex cases, etc. They may also facilitate ongoing training.
Doula Payment Model	Initially, SisterWeb doulas were paid approximately $1600 per client (originally estimated to be about $25/h, without benefits). This covered prenatal visits, labor and birth support, and postpartum visits, attendance at professional development trainings, and mentorship sessions. SisterWeb has since transitioned all doulas to an hourly employment model with benefit eligibility in order to make community doula work more sustainable for their doulas. All doulas are guaranteed to work at least 30 hours per week for SisterWeb.
Workforce Development	SisterWeb’s workforce development program supports its community doulas on their career paths as professional birth workers by providing monthly professional development sessions and mentorship built-in to their cohorts. Trainings cover a variety of topics including trauma-informed pelvic exams, breastfeeding and lactation, and adverse childhood experiences, all with an anti-racism and anti-bias lens.
Champion Dyad Initiative	The Champion Dyad initiative fosters the support of one or two “champions” at each hospital who partner with a SisterWeb staff member. The goal of this close partnership is to advance quality improvement and ensure that birthing people of color receive fair and equitable treatment during their births and pregnancies. The partners in a Champion Dyad have close, direct access to each other with the goal of being able to bring issues, changes in process or hospital policy, or any other concerns to the forefront quickly and within a trusting relationship; Champion Dyad members can then act as conduits to the rest of their hospital team or SisterWeb doulas. Currently, this initiative is being implemented in the San Francisco hospital labor and delivery units serving the vast majority of Black and Pacific Islander pregnant people who give birth.

## Methods

We conducted a process evaluation of two of SisterWeb’s three programs, Kindred Birth Companions (KBC) and M.A.N.A. Pasefika, between 2018 and 2020. As the evaluation was funded to examine these two programs, the process evaluation did not include SisterWeb’s third program. The purpose of the process evaluation was to monitor the implementation of the programs—whether they were being implemented as designed, working well, accessible to clients and doulas, and supported by hospital staff. The full process evaluation included both qualitative and quantitative components; this paper reports on the qualitative component, which focused on implementation-related barriers and facilitators. All methods and results are reported according to the Consolidated Criteria for Reporting Qualitative Research (Tong et al., 2007).

### Conceptual Framework

The process evaluation was guided by the Equitable Evaluation Framework™, ensuring that the evaluation is designed to advance equity, pay attention to historical, structural, and cultural context, and is rooted in collaboration (Luminaire Group et al., 2017). By utilizing this approach, we went beyond traditional process evaluation methods to examine external, systemic factors influencing SisterWeb’s ability to implement their programs. In line with this framework, we created a diverse research team including partners based at local academic institutions and organizational leaders from SisterWeb, and collaborated closely on the evaluation design, execution of evaluation activities, data collection and interpretation, and dissemination of findings. The Committee for the Protection of Human Subjects as the University of California, Berkeley approved the study protocol.

Seven trained members of the study team, including authors CM, AMG, and SA, conducted individual in-depth qualitative interviews with five groups: SisterWeb doulas, doula mentors, leadership team members, clients, and external stakeholders. Two members of the team with no previous qualitative interviewing experience participated in a training led by AMG and CJM, conducted practice interviews, and received feedback from SA and JA; all other interviewers had extensive experience conducting qualitative interviews. Due to the participatory nature of this study, some interviewers were known to participants prior to data collection, however, no additional information was provided to participants about the interviewer prior to the interview. Interviewers and clients were concordant by race and gender, while interviewers and doulas, mentors, and leadership were concordant by gender only. Interviewers and external stakeholders were not matched by race or gender. All participants were invited to participate via email except for clients, who were invited to participate in-person by SisterWeb’s Doula Coordinator. All individuals in each group (e.g., doula, client, etc.) were invited to participate in interviews. While some individuals (n = 23) were unable to be interviewed, mainly due to nonresponse to recruitment emails, we were able to interview everyone who wanted to participate during the study period.

Interviews (n = 46) occurred between September 2019 and July 2020. Interviews with doulas (n = 6) and mentors (n = 2) explored their experiences providing care and mentorship, and their perspectives regarding barriers to providing care and the sustainability of their work with SisterWeb. SisterWeb leadership interviews (n = 4) focused on program development, barriers and facilitators to program implementation, and any programmatic changes that have occurred since the organization’s launch. In client interviews, we investigated experiences with and reasons for program participation; we conducted interviews at two timepoints: after the first or second prenatal visit (n = 14) and after at least one postpartum visit (n = 9). Finally, we interviewed external stakeholders (n = 11) about their relationships and experiences with SisterWeb to further identify barriers and facilitators to program implementation; stakeholders included SisterWeb’s partners at local hospitals and city and social service agencies. All interviews were conducted via phone, videoconference, or in-person, and in English; interviewees may have spoken other languages, including Spanish and Samoan.

Interviews were transcribed verbatim and lasted an average of 63 min. Members of the study team reviewed transcripts for errors and removed identifiers. We analyzed the data utilizing a Rapid Assessment Process (RAP), a team-based, rigorous analytic approach. RAP includes iterative analysis, additional data collection, and data triangulation in order to develop preliminary, actionable findings; the rapid turnaround of data findings makes this approach well-suited for qualitative research informing developing interventions as it allows for concurrent intervention modifications (Beebe, 2008). Despite the “rapid” nature of this analytic method, the action-oriented approach utilizes strategic data collection to facilitate data reduction and synthesis for time-sensitive projects. Our analysis involved a five-step process (Hamilton, 2013). First, we developed summary templates with neutral domains matching interview questions for each participant group. Interviewers used these templates to create field notes in the form of an interview summary for each interview immediately or soon after completion. After interview transcript cleaning, we updated the summary transcripts to capture any data missed in the original summary. Once finalized, we transferred the transcript summaries to individual-level data matrices for each participant group to facilitate synthesis of key data across participants and domains. We then created group-level memos, summarizing main findings for each domain across participants. Lastly, we reviewed group-level memos and mined individual transcripts and transcript summaries for emergent themes. We did not employ participant checking, however, we clarified programmatic and organizational questions with SisterWeb leadership to gain a more comprehensive understanding of the data. Below, we present the findings from the qualitative analysis, organized by key barriers and facilitators to implementation.

## Results

### Facilitators

#### Clear Organizational Vision and Mission

From inception, SisterWeb’s leadership outlined a vision and mission for building a community of professional birth workers and educators from and serving Black, Pacific Islander, and Latinx birthing people in San Francisco. The vision and mission served as a clear, uniting force that aligned SisterWeb staff, doulas and mentors, and community partners, which ultimately helped propel their work forward and implement their programs even when facing challenges.

“I think SisterWeb has done a really good job of making sure that anything that they apply for or get funding for is in line with their mission and vision, and not compromising who they are or their integrity and what kind of care they want to provide.”

-Stakeholder

Our findings revealed that SisterWeb staff were mission-driven and deeply committed to addressing maternal health inequities in their community. Stakeholders, including healthcare providers and public health professionals, were impressed with Sisterweb’s authenticity, integrity, transparency, and commitment to their mission. Further, doulas and mentors strongly identified with the organization’s mission and passion for addressing maternal health disparities and cited this as a key reason that they enjoyed working with SisterWeb.

#### Unique Doula Cohort Model

One aspect of SisterWeb’s program is the doula cohort model, where each client is assigned a cohort consisting of two or three doulas and a mentor. SisterWeb chose to use a cohort model based on their experiential knowledge and review of previous successful doula programs. They hypothesized that the cohort model would increase the sustainability of doula work, as doulas can rely on each other during family emergencies and illness, provide a way for doulas to balance their doula responsibilities with their personal and other professional commitments, and allow doulas to work in shifts during long births. SisterWeb incorporated mentors into the cohort to provide doulas with professional and emotional support, guidance, and coaching to support their confidence and ability to manage clients’ pregnancy and birthing needs, a benefit that doulas described wishing they had earlier in their careers.

I feel like [being a SisterWeb doula] has been life-changing… I have gained information and connections and mentorship that I would not have had, had I not been a part of this program.

-Doula

This model proved to be an essential ingredient of SisterWeb’s ability to implement their programs successfully. Interviews revealed it was effective at providing increased support for clients by ensuring they always had a doula available while also allowing doulas to share workloads and avoid burnout.

“[Having two doulas] makes me feel, you know, confident about the support that I'm getting from this program, that I'm going to have at least one person from this program to be there.”

-Client

“If I was, like, a doula on my own I would definitely need a partner. But the cohort system like, it kind of gives you like a built-in backup. You always know that someone has your back like if it goes too long, if you need a break, if your work gets, you know, you got work conflict like for prenatals and the birth. Like it’s honestly like, a huge, huge, huge relief.”

-KBC Doula

#### Established Partnerships and Positive Reputation

Relationships with outside organizations and stakeholders, particularly at hospitals, are crucial to the delivery of doula care, especially the integration of doulas into labor and delivery units. Partnerships were also integral to SisterWeb’s work to ensure appropriate referrals to their program.

“Championing our work within the hospital [contributes to] the longevity of fixing the problems…And the money again, it'd be easy to say that's the [most important] thing. Do we need it? Yes. To run it? Yes. But the real change is happening with the clients in the hospitals. It's like the secret power.”

-Leader

We found that, overall, SisterWeb was positively regarded among the community stakeholders that they work with, and that this was key to launching and implementing their doula programs. SisterWeb leaders were strong at maintaining strategic partnerships within the community and with local institutions. One example of this is the Champion Dyad Initiative, a partnership between representatives at local hospitals and SisterWeb that was co-developed with key stakeholders, including Expecting Justice. As one leader said, Champion Dyads allows SisterWeb to “discuss what's going well with the doulas working at that particular hospital and what isn't going so well.” While we found there were some areas for improvement, such as streamlined communication between SisterWeb and referral sites, overall, these positive, established partnerships were critical to SisterWeb’s success. Importantly, in building these relationships, SisterWeb was able to leverage the advocacy and legacy of other birth justice organizations and doulas in San Francisco.

#### Unmet Need for Community Doula Services Among Clients

Overall, clients described a genuine need for the program and felt it provided an important service to their community. Importantly, some clients indicated a need for doula support based on their previous negative pregnancy and birth experiences, while others described needing extra support in addition to their support system. Notably, a few clients expressed concerns about the high rates of negative birth outcomes among women in their community, and many were drawn to the program to have an advocate during the birthing process and because they desired racially-concordant, community-focused support.

"Support system as far as like somebody that's communicating to me, somebody to be there for my labor... Especially with this being my first child. Like I just need a lot of – I won't say a lot of attention, but I just need a lot of people there worried about how I'm feeling and what I'm going through and stuff at the moment.”

-Client

Clients were very satisfied with the services provided by SisterWeb. Clients offered few suggestions for improvement and reported they would recommend SisterWeb to others. This positive evaluation was with respect to not only the services directly provided by doulas (e.g., prenatal and postpartum visits, labor and delivery support) but also the entire process of becoming enrolled in the program, such as the intake and doula matching processes. Overall, SisterWeb’s model of services was extremely well-received by clients.

“The attention and everything was great. The doulas there made me feel really comfortable doing something that I've never done before... I recommended the program to a friend that's currently pregnant, and she was accepted. So, she'll be doing it as well yeah. Very, very happy that it worked out.”

-Client

### Barriers

#### Simultaneously Launching a Doula Program and a Doula Organization

From the initial funding, SisterWeb was tasked with creating two programs to provide community doula care to Black and Pacific Islander pregnant people. SisterWeb as an organization did not exist at that point, and in order to launch the programs, they needed an organizational structure. Thus, SisterWeb simultaneously had to formalize as an organization, including obtaining a fiscal sponsor and onboarding staff, while also developing and implementing the specific doula programming. Their initial funding and planned timeline for the launch of the programs did not fully account for this complexity, which meant they were operating with limited time, funding, and administrative support.

“We did not have the time or the designated funding or staffing to create a planning process. So, it was like telling somebody, here's money to build a house, but we don't have time to hire an engineer or architect. So, carpenter, take it away. And you gotta start construction tomorrow.”

-Leader

These limitations led to organizational challenges including slower internal and external communications and processes and disparate capacity for growth among SisterWeb’s programs, especially the Pacific Islander-serving program, which has unique community engagement and educational needs. These included a limited awareness about doula care and its benefits within the San Francisco Pacific Islander community, a potential preference for group- and community-based care instead of one-on-one support, and a need for materials to be translated into the many languages spoken by Pacific Islander people.

“I feel like the needs of our program are also a lot different, just because our community don't know a lot about doulas and having birth support… Pacific Islanders have like the highest rates of preterm birth in San Francisco, I feel like what we're doing right now is a lot more having to educate people about why Pacific Islander doulas are needed and what exactly that we do.”

-M.A.N.A Pasefika Doula

#### The Doula Payment Model did not Meet Organizational Needs and Values

SisterWeb initially used a per-birth, contractor payment model, wherein doulas are paid a lump-sum per birth supported. This payment model was due to the quick timeline in which the initial funding was awarded. SisterWeb leaders had a week to submit the proposal; they swiftly developed a payment model, combining popular approaches at the time, including private doula care models and those used by existing community doula organizations. However, leaders realized, from inception, that this payment approach was flawed and led to doulas not being paid an amount commensurate with their effort and hours worked, given that SisterWeb doulas were often providing services beyond what typical, private doulas offer. SisterWeb doulas work in cohorts of two or three doulas and go above and beyond for their clients to ensure they are supported in their pregnancies and births; doulas worked more hours than anticipated while receiving a fixed amount of pay, regardless of time spent with their clients, that they then had to split among cohort members. Furthermore, in addition to direct client services, doulas participated in regular professional development workshops and mentorship sessions.

“So, [doulas] get paid for the birth but don't get paid for a lot of the other stuff in between… we're asking [doulas] to do a lot. We're not just asking you to go sit and do your prenatal. We're asking you to track information, come to these meetings, come to your monthly business meeting, your monthly professional development.”

-Doula Coordinator

The initial payment model undermined SisterWeb’s program needs and values given their goal of a sustainable employment model and livable wages for their doulas. Notably, nearly all doulas worked multiple jobs to make ends meet and several were caretakers of children and family members. An in-depth examination of SisterWeb’s different compensation models has been published elsewhere (Gomez et al., 2021).

#### Diverse Clientele with Differing and Unique Needs

As described earlier, SisterWeb’s programs that were the focus of this evaluation provide community doula care to two distinct racial/ethnic communities—Black and Pacific Islander pregnant people. A hallmark of community doula care is the provision of culturally relevant and concordant services. As a small and nascent organization, SisterWeb faced some challenges in simultaneously providing services for two distinct cultural groups, each with their own unique needs. Because of this, and given there are more births among Black people than Pacific Islander people in San Francisco, the programs experienced unequal growth. The program serving Pacific Islander pregnant people launched later than the program for Black people and was more challenging to grow, in part because SisterWeb needed to become a trusted organization within the Pacific Islander community. Notably, SisterWeb’s leadership circle has long-standing relationships with Black or Latinx communities in San Francisco, which likely contributed to the development of those respective programs.

“[We need to] build trust in the community, you know? People aren't going to just be like, oh, you're doulas. Oh, you're Pacific Islanders? Okay, you can come to this really intimate event in my life.”

- M.A.N.A. Pasefika doula

## Discussion

This process evaluation documented the barriers and facilitators that a community-based doula organization serving Black and Pacific Islander birthing people in San Francisco experienced as they launched their organization and programs. The factors that facilitated the implementation of SisterWeb’s programs, such as a clear, shared vision and mission, are consistent with the literature on community-based program implementation (Issel & Wells, 2017). Similar to our findings, strong engagement of a broad range of stakeholders and partners has been cited as central to the development and implementation of other community-based doula programs (HealthConnect One, 2014). Program planning models emphasize the importance of ensuring that programs appropriately meet the needs of the communities they serve (Fernandez et al., 2019; Issel & Wells, 2017). We found that SisterWeb’s clients felt that the services addressed an important, unmet need in their communities, which facilitated program implementation. However, our findings also suggested that additional efforts are needed to ensure that the unique needs of the Pacific Islander community are met. With respect to barriers, limited time, funding, and technological and administrative support, particularly in the start-up phase of a program, have been cited as challenges to developing programs (Sherman et al., 2017). We found that SisterWeb, having to simultaneously launch their doula programs and formalize as an organization, experienced all of these limitations, which led to implementation challenges. Ensuring livable wages for community doulas has similarly been identified as a key challenge to the implementation of Medicaid coverage of doula care in Oregon and Minnesota, the two states that have this coverage (Bey et al., 2019).

Notable strengths of this study include the partnership between our research team and the community doula organization, which guided all of our work, including the study design. Specifically, this partnership enabled us to gain intimate knowledge of the organization’s inner workings, which was beneficial in focusing our data collection efforts. The qualitative focus of our study and the inclusion of organizational leaders, clients, doulas, and stakeholders in our sample, allowed us to gain a nuanced and holistic understanding of the facilitators and barriers to implementing culturally congruent community doula programs, a topic for which limited literature exists. Limitations include the geographical context, which has financial and structural implications for the operation of the organization, and lack of data from clients and doulas who left the organization. Future research should examine the implementation processes of community doula programs in other geographical contexts.

### Implications for Policy and Practice

Community-based, culturally congruent models of doula care are receiving increasing attention across the country as an intervention to reduce racial disparities in poor maternal and infant health outcomes. For example, at the present time, we are aware of at least 7 other pilot programs across California to provide community doula care that are in various stages of implementation (California Department of Public Health, n.d.; Frontline Doulas, n.d.). We believe our findings offer important considerations for the implementation of current and future community doula programs, their funders, and the hospitals where community doulas work.

Launching a community doula *organization* is different than launching a community doula *program*. Specifically, building a robust, nonprofit, mission-driven organization from the ground up, particularly when there are few similar organizations as examples, requires adequate time, effort, and funding outside of program development. In order to support grassroots organizations like SisterWeb meet its goals, funders should consider the necessary inputs for building an organization up from scratch, and provide funds and time needed for the critical planning phase. Funders should also consider the need for program evaluation and provide funding for researchers to engage in equitable evaluation. In doing so, researchers can work with developing community doula organizations to generate valuable data to inform in-real-time programmatic improvements and contribute to a shared best-practice guide for future community doula programs.

Considerable attention should be paid to doula payment models to ensure sustainable and livable wages, particularly for community doulas. Community doulas often provide services at low or no cost in order to increase access for those who need these services most, creating substantial barriers to the sustainability of this work. Contractor payment models based on births may perpetuate inequity in employment conditions for community doulas and access to community doula services for birthing people of color. In order to increase the sustainability of community doula work, we suggest organizations employing community doulas look beyond private doula payment models to hourly models of employment with the opportunity for benefits. Furthermore, policymakers should engage community doulas in advocacy efforts for Medicaid coverage of doula care, a potential, albeit logistically complicated, pathway to increasing equitable labor conditions for community doulas.

Community doula programs and services must also be tailored to be culturally relevant to the communities served, and, as such, should be rooted in lived-experience, respect for culture, and community voice. Our findings suggest that community doula programs may benefit from time and funds to conduct a thorough needs assessment, led by and in partnership with their target communities, to determine community needs. However, efforts to do this should be inclusive of the communities to be served, including providing specific, culturally relevant training, engaging community members in the planning phase, and employing community leaders. Lastly, community doula organizations will be most successful when they are led by racially- and culturally-concordant community members, who share lived experiences with their clients and possess a deep knowledge of their needs.

Our findings suggest strategic partnerships are critical to the success of community doula programs. SisterWeb leaders described their partnerships as a cornerstone of their program, providing them with opportunities to integrate into the hospitals where their clients give birth and receive referrals to the program. As such, stakeholders at hospitals where doulas work should partner with community doula organizations to advance health equity for birthing people of color and with low-incomes in meaningful, concrete, and measurable ways.

## Data Availability

The data generated from this study are not publicly available.
